# Quercetin prevents progression of disease in elastase/LPS-exposed mice by negatively regulating MMP expression

**DOI:** 10.1186/1465-9921-11-131

**Published:** 2010-09-28

**Authors:** Shyamala Ganesan, Andrea N Faris, Adam T Comstock, Sangbrita S Chattoraj, Asamanja Chattoraj, John R Burgess, Jeffrey L Curtis, Fernando J Martinez, Suzanna Zick, Marc B Hershenson, Uma S Sajjan

**Affiliations:** 1 Department of Pediatrics and Communicable Diseases, University of Michigan, Ann Arbor, MI, 48109 USA; 2 Department of Internal Medicine, University of Michigan, Ann Arbor, MI, 48109 USA; 3 Department of Family Medicine, University of Michigan, Ann Arbor, MI, 48109 USA; 4 Department of Molecular and Integrative Physiology, University of Michigan, Ann Arbor, MI, 48109 USA; 5 Department of Foods and Nutrition, Purdue University, West Lafayette, IN, 47906 USA

## Abstract

**Background:**

Chronic obstructive pulmonary disease (COPD) is characterized by chronic bronchitis, emphysema and irreversible airflow limitation. These changes are thought to be due to oxidative stress and an imbalance of proteases and antiproteases. Quercetin, a plant flavonoid, is a potent antioxidant and anti-inflammatory agent. We hypothesized that quercetin reduces lung inflammation and improves lung function in elastase/lipopolysaccharide (LPS)-exposed mice which show typical features of COPD, including airways inflammation, goblet cell metaplasia, and emphysema.

**Methods:**

Mice treated with elastase and LPS once a week for 4 weeks were subsequently administered 0.5 mg of quercetin dihydrate or 50% propylene glycol (vehicle) by gavage for 10 days. Lungs were examined for elastance, oxidative stress, inflammation, and matrix metalloproteinase (MMP) activity. Effects of quercetin on MMP transcription and activity were examined in LPS-exposed murine macrophages.

**Results:**

Quercetin-treated, elastase/LPS-exposed mice showed improved elastic recoil and decreased alveolar chord length compared to vehicle-treated controls. Quercetin-treated mice showed decreased levels of thiobarbituric acid reactive substances, a measure of lipid peroxidation caused by oxidative stress. Quercetin also reduced lung inflammation, goblet cell metaplasia, and mRNA expression of pro-inflammatory cytokines and muc5AC. Quercetin treatment decreased the expression and activity of MMP9 and MMP12 *in vivo *and *in vitro*, while increasing expression of the histone deacetylase Sirt-1 and suppressing MMP promoter H4 acetylation. Finally, co-treatment with the Sirt-1 inhibitor sirtinol blocked the effects of quercetin on the lung phenotype.

**Conclusions:**

Quercetin prevents progression of emphysema in elastase/LPS-treated mice by reducing oxidative stress, lung inflammation and expression of MMP9 and MMP12.

## Background

Chronic obstructive pulmonary disease (COPD) is a heterogeneous disorder characterized by small airway inflammation/fibrosis, mucus plugging and emphysema. COPD is the fourth leading cause of death worldwide and the prevalence is predicted to rise in the next two decades [1].

Although the cellular and molecular mechanisms of COPD pathogenesis are not well known, oxidative stress, chronic inflammation and an imbalance of proteases and antiproteases are thought to play key roles in development and progression of the disease [1]. Therefore, a treatment with antioxidant and anti-inflammatory properties could be beneficial in preventing or slowing the progression of lung disease in COPD.

Inhalation of cigarette smoke and other environmental exposures can stimulate resident alveolar macrophages and lung epithelial cells to generate reactive oxygen species (ROS) and reactive nitric oxide species (RNS) in excess, thereby disturbing the oxidant to antioxidant balance, resulting in oxidative stress [2-4]. ROS and RNS stimulate the production of a number of host mediators, some of which can attract neutrophils, macrophages and other inflammatory cells to the lungs. Recruited inflammatory cells and epithelial cells produce matrix metalloproteinases (MMPs), thereby increasing protease activity in the lungs. MMPs, in turn, degrade alveolar walls, leading to enlargement of airspace and development of emphysema. In addition, chronic inflammation causes remodeling of the airways, including goblet cell metaplasia, mucus plugging and airway wall thickening.

The histone deacetylases (HDACs) are transcriptional repressors which have been implicated in the resolution of inflammation as well as the regulation of MMP expression [5]. In COPD patients, reductions in the expression of HDAC2 and HDAC5 correlate with disease severity and increased IL-8 expression [6]. Similarly, the type III HDAC Sirt1, which regulates MMP expression, was also found to be downregulated in COPD [7].

Lipopolysaccharide (LPS), a cell wall component of gram negative bacteria, is a potent inflammatory molecule and is present in appreciable amounts in cigarette smoke. It is also an active component in environmental and occupational exposures associated with the development of COPD [8-10]. Experimental inhalation of LPS evokes pulmonary and systemic inflammation in healthy human subjects [11,12]. Chronic exposure of experimental animals causes emphysema, goblet cell metaplasia, and airway wall thickening. These alterations persist up to four to eight weeks following LPS administration [13,14]. Recently, we demonstrated that mice exposed to a combination of LPS and elastase once a week for 4 weeks display COPD-like features including widespread lung inflammation, goblet cell metaplasia, increased lung volume, emphysema and decreased elastic recoil [15]. These changes persisted up to 8 weeks after cessation of exposure to elastase and LPS. These mice were also found to be more susceptible for rhinovirus infection.

Quercetin is a 3,3',4',5,7-pentahydroxyflavone found in many plants. Based on its polyphenol structure, quercetin has potent antioxidant effects, combining with free radical species to form considerably less reactive phenoxy radicals [16,17]. Quercetin also has anti-inflammatory effects, inhibiting lipid, protein tyrosine and serine/threonine kinases by its capacity to compete with the binding of ATP at the nucleotide binding site [18]. Previously, we demonstrated that quercetin inhibits TNF-α stimulated IL-8 expression at the transcriptional level in airway epithelial cells and decreases airways hyperresponsiveness in cockroach allergen-sensitized and challenged mice, a model of allergic airways disease, at a dose of 0.6 mg per day (approximately 30 mg/kg body weight) [19]. Quercetin was also shown to suppress eosinophilic inflammation in ovalbumin-sensitized and -challenged mice at a dose of 10 mg/kg body weight [20]. Further, quercetin decreases the expression of MMP9 stimulated by TNF-α in epidermal cells [21]. Based on these observations, we hypothesized that quercetin reverses oxidative stress and inhibit MMP production, perhaps by increasing the expression of the histone deacetylase Sirt-1, thereby preventing the progression of lung disease in COPD. To test this hypothesis, we treated elastase/LPS exposed mice with quercetin (10 mg/kg body weight), and examined oxidative stress, inflammation and expression of MMP9, MMP12 and SIRT-1 in the lungs. We also determined the effect of quercetin on the histone acetylation of the MMP9 and MMP12 promoters by chromatin immunoprecipitation assay.

## Methods

### Animals and treatment

Eight to ten weeks old C57BL/6 mice (Charles River Laboratories International Inc., Wilmington, MA) were exposed to elastase and LPS for four consecutive weeks as described previously [15]. Animals were exposed by the intranasal route to 1.2 U of porcine pancreatic elastase (Elastin Products, Owensville, MO) on day one and 7 μg (approximately 70 endotoxin units) of LPS from *E. coli *O26:B6 (Sigma-Aldrich, St. Louis, MO) on day four of the week for four consecutive weeks. Control mice were exposed to PBS. Seven days after the last exposure to LPS, mice were orally gavaged with 300 μl of 50% propylene glycol (vehicle) or 0.2 mg of quercetin (10 mg/kg body weight; Sigma-Aldrich, St. Louis, MO) dissolved in 50% propylene glycol, once a day for 10 days. We chose this dose of quercetin based on previous studies in which quercetin at 10 mg/kg body weight reduced airways responsiveness and lung inflammation in an allergic mouse model of asthma [19,20]. In some experiments, mice were treated intraperitoneally with 100 μl PBS or PBS containing sirtinol (0.5 mg/kg body weight, Calbiochem, Gibbstown, NJ) along with quercetin or vehicle for 10 days. Mice were sacrificed 1 h after the last quercetin treatment. In some experiments, mice were examined 7 and 17 days after the last exposure to LPS without any treatment in order to examine the progression of emphysema after cessation of exposure to elastase/LPS. Unexposed mice treated with vehicle or quercetin (10 mg/kg body weight) were used as negative controls. All experiments described herein were approved by the Animal Care and Use Committee of the University of Michigan.

### Measurement Of Lung Elasticity

Mice Were Anesthetized By Intraperitoneal Injection Of Ketamine (2.5-5 Mg/100 G Body Weight) And A Steel Cannula Was Inserted Into The Trachea And Connected To A Miniature Computerized Flexivent Ventilator (Scireq, Montreal, Quebec, Canada). Sodium Pentabarbitol (2 Mg/100 G Body Weight) Was Also Given To Provide Further Sedation And Allow Stabilization On The Ventillator. To Determine Elastic Recoil, Lungs Were Gradually Inflated To 30 Cm H_2_O And Pressure And Lung Volume Measured Continuously During Inflation And Deflation Of The Lungs. Static Elastance And Compliance Were Recorded By Inflating The Lungs To Full Capacity.

### Lung Histology And Morphometry

Lungs Were Perfused With 20 Mm Edta And Inflation-Fixed With 10% Buffered Formalin, And Embedded In Paraffin. Five Micron Thick Sagittal Sections Were Stained With Hematoxylin And Eosin (H & E) Or Periodic-Acid Schiff'(Pas) Reagent. Alveolar Chord Length Was Determined Using Sagittal Sections Obtained At 5 Mm Intervals Through The Length Of The Lungs, And Diameter Of The Airspaces Was Measured In Random Areas Using Nih Image J Analysis Software [15].

### Bronchoalveolar Lavage (Bal)

Mice Were Euthanized And Lungs Were Lavaged With Pbs. Bal Fluid Was Centrifuged And The Supernatant Was Collected For Determination Of Mmp Levels. Total And Differential Cell Counts In Bal Fluid Were Determined As Described Previously [15,22].

### Lung Cytokine Levels

After Relevant Treatment, Mice Were Euthanized, Lungs Were Collected, Homogenized In Pbs Containing Complete Protease Inhibitors And Centrifuged (Roche, Indianapolis, In). Cytokine Protein Levels In The Lung Homogenate Supernatants Were Measured Either By Elisa (R & D Systems, Minneapolis, Mn) Or Multiplex Immunoassay (Biorad, Hercules, Ca) [15,22].

### Alveolar Macrophage Cell Culture

Murine Alveolar Macrophages (Crl-2019, American Type Culture Collection, Manassas, Va) Were Cultured In Rpmi1640 Supplemented With 10% Fetal Bovine Serum, Penicillin (100 Units/Ml), Streptomycin (100 μG/Ml), 1% Glutamine And 0.01% β-Mercaptoethanol. To Determine The Effect Of Quercetin On Mmp Expression, Cells Were Seeded In 6 Well Plates And Grown For 24 H. Cells Were Then Exposed To Cell Culture Media Containing 1 Ng/Ml Lps For 8 Hours A Day For Three Days. In Between Exposures To Lps, Cells Were Maintained In Cell Culture Media Alone. Cells Were Then Shifted To Serum-Free Media Containing Quercetin Dihydrate Or Dmso, Incubated For 24 H, And Media And Cells Were Harvested. Cells Exposed To Media Alone Instead Of Lps Were Used As Negative Controls.

### Gelatin Zymography

Mmp Activity Was Determined By Gelatin Zymography As Described.[23]. Briefly, Equal Volumes Of Bal Supernatant Or Conditioned Cell Culture Media Was Incubated With Non-Reducing Sample Buffer And Subjected To Electrophoresis On 8% Polyacrylamide Gels Impregnated With 0.1% Gelatin. Gels Were Washed With 1% Triton X-100, Developed In Tris Buffer Containing 10 Mm Cacl_2 _And 5μM Zncl_2 _And Stained With 0.5% Coomassie Blue.

### Measurement Of Plasma Quercetin Levels

Mice Were Sacrificed And Blood Was Collected By Cardiac Puncture In Tubes With Anticoagulant, Centrifuged And Plasma Was Collected. Levels Of Quercetin In Plasma Were Determined By Hplc As Described Previously [19].

### Chromatin Immunoprecipitation (Chip) Assay

Chip Assays Were Performed With A Chip-It Kit (Active Motif, Carlsbad, Ca) Following The Manufacturer'S Instructions. Briefly, Cells Were Fixed, Lysed And Chromatin Was Subjected To Enzymatic Shearing. Chromatin Fragments Of 100-1000 Bp Were Immunoprecipitated With An Antibody To Acetylhistone H4 Antibody. Chip And Input Dna Were Purified And Subjected To Qpcr Using Primers Specific For The Nf-κB Binding Site In The Mmp9 And Mmp12 Promoters. Qpcr Conditions Were As Follows: 95°C For 15 Minutes; 95°C For 10 Seconds, 60°C For 30 Seconds, 72°C For 30 Seconds, Repeated For 50 Cycles, 72°C For 10 Minutes. The Primers Used For Qpcr Were Mmp9: Sense 5'-TTTAAACAGAAGAGGAAGGAT AGTGC-3' And Antisense 5'-CCTGATAGAGTCTTT GACTCAGCTTC-3'; Mmp12: Sense 5'-TTGCTGAATCATTTCATGGC-3' And Antisense 5'-AGTGCATAGGTATG TGAATGGG-3'.

### Quantitative Pcr

Expression Of *Mmp9*, *Mmp12*, *Sirt1*, Inducible Nitric Oxide Synthase (*Inos*), Heme Oxygenase (*Hmox)-1*, And *Muc5Ac *Was Determined By Qpcr. All Pcr Reactions Were Performed In An Eppendorf Mastercycler (Westbury, Ny) And Gene Expression Was Quantified Using The Comparative Ct Method.

### Western Blotting

Nuclear Proteins Were Resolved By 7.5% Sds-Polyacrylamine Gel Electrophoresis, Proteins Transferred To Nitrocellulose Membrane And Probed With Antibody To Sirt1 And β-Actin (Santa Cruz Biotechnology, Santa Cruz, Ca). Specific Bands Were Quantified By Densitometry Using Nih Imagej And Expressed As A Ratio Of Sirt1/B-Actin Which Is Normalized To Untreated Control Mice.

### Lipid Peroxidation

The Amount Of Lipid Peroxidation Products In The Lungs Was Assayed As Thiobarburtic Acid Reacting Substances (Tbars) (Cell Biolabs, San Diego, Ca) Following Manufacturer'S Instructions.

### Statistical Analysis

Statistical Analysis Of Significance Was Calculated By One-Way Analysis Of Variance Followed By Tukey'S Post Hoc Test, Anova On Ranks With Dunn'S Post Hoc Analysis Or By Mann-Whitney Test As Appropriate. Results Represent Mean ± Sd Or Sem, Or Range Of Data With Median.

## Results

### Effect of quercetin on elastase/LPS-induced emphysema

**Figure 1:**
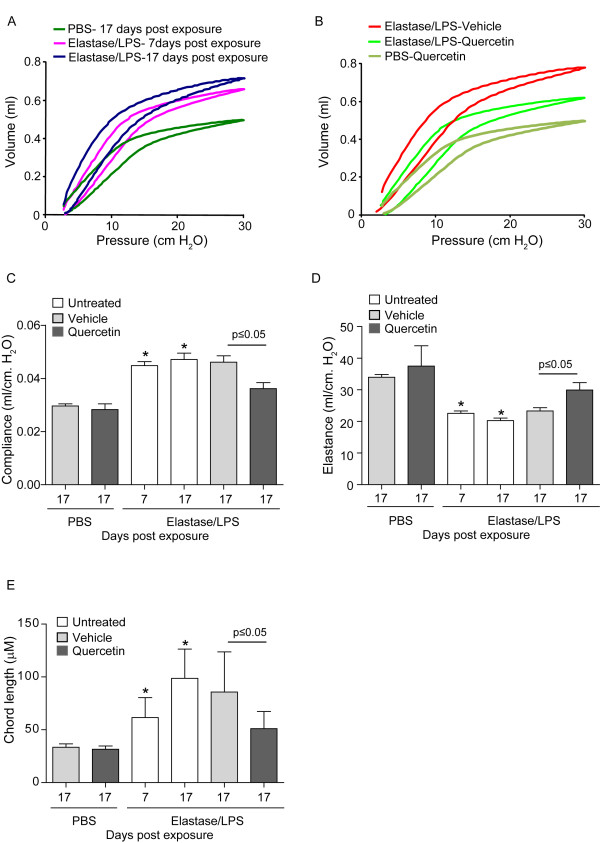
**Quercetin partially improves lung function in elastase-LPS exposed mice**. Mice were anesthetized and pressure-volume relationships, compliance and elastance were measured using flexivent system. Chord length was determined by morphometry. Seven days after the last exposure to elastase/LPS, untreated mice showed a leftward and upward shift in the pressure-volume (PV)-loop (A) compared to PBS-exposed vehicle treated mice, indicative of a loss of elastic recoil. Seventeen days after the last exposure to elastase/LPS, this curve shifted further, indicating progression in the loss of elastic recoil. Quercetin treatment for 10 days prevented progression in loss of elastic recoil in elastase/LPS-exposed mice but had no effect on PBS-exposed mice (B). Each of these mice was examined 17 days after the last elastase/LPS treatment. Elastase/LPS-exposed mice treated with quercetin also showed increased elastance (C), decreased compliance (D) and decreased alveolar chord length (E) compared to vehicle treated mice. Representative PV curves from 5 to 6 mice from each group are shown in A and B. Data in C, D and E represent mean and SD calculated from 6 animals per group (*different from PBS group, p≤0.05 one-way ANOVA; † different from vehicle treated mice, p≤0.05 Mann-Whitney test).

As observed previously [15], mice at seven days after the four-week exposure to elastase/LPS showed an upward and leftward shift compared to control mice, demonstrative of reduced elastic recoil (Figure 1A). Because mice receiving quercetin or vehicle for 10 days were studied a total of 17 days after the last exposure to elastase/LPS, we also measured the lung function of untreated mice at this time point. The volume-pressure curve was shifted further to the left, indicating further progression of emphysema after cessation of exposure to elastase/LPS. This shift may be due to persistence of oxidative stress and MMP activity even after cessation of exposure to elastase/LPS. This situation is analogous to the further progression of emphysema in COPD patients even after cessation of smoking [24].

Next, we examined the lung function of mice treated with quercetin (0.2 mg) or vehicle for 10 days starting one week after the four-week course of elastase/LPS-treatment. Compared to vehicle, mice receiving quercetin showed a rightward and downward shift in their volume-pressure curve (Figure 1B). Shifts in the pressure-volume loops were accompanied by appropriate changes in elastance and compliance (Figures 1C and 1D). Finally, compared to vehicle, quercetin treatment was associated with a reduction in alveolar chord length (Figure 1E). However, quercetin treatment did not completely reverse the emphysematous changes caused by elastase/LPS. Quercetin treatment did not affect any of these measurements in the lungs of mice exposed to PBS. Together, these data suggest that quercetin treatment prevented further progression of emphysema after elastase/LPS treatment rather than stimulating the regeneration of degraded alveoli.

### Quercetin decreases oxidative stress in elastase/LPS-exposed mice

**Figure 2:**
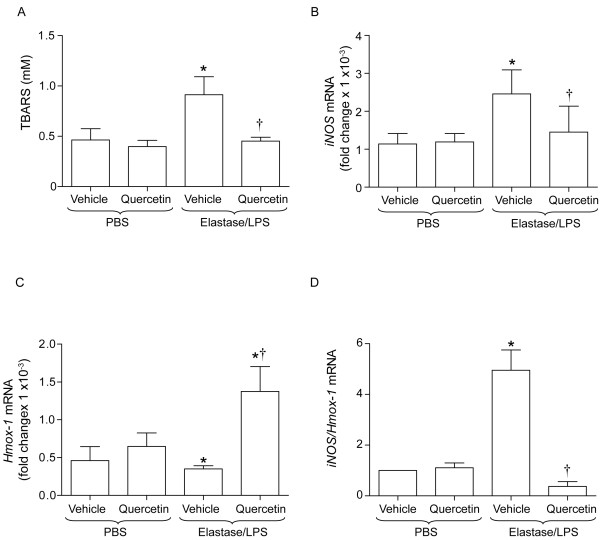
**Quercetin treatment reduces TBARS and increases *Hmox-1 *expression in elastase/LPS exposed mice**. Mice exposed to PBS or elastase/LPS were orally gavaged with 0.2 mg of quercetin or vehicle daily for 10 days and sacrificed on the last day of quercetin treatment. A to D. Elastase/LPS exposed mice show increased levels of TBARS, increased *iNOS *mRNA levels, decreased *Hmox-1 *mRNA and increased ratio of *iNOS*/*Hmox-1 *compared to PBS exposed mice treated with either vehicle or quercetin. Quercetin treatment reduces TBARS, increases *Hmox-1 *mRNA and decreases ratio of *iNOS*/*Hmox-1 *in elastase/LPS-exposed mice. Data represent mean and SEM (n = 10-14, *different from PBS/vehicle and PBS/quercetin group, p≤0.05; † different from vehicle treated elastase/LPS-exposed mice, p≤0.05 one-way ANOVA).

To determine the mechanism by which quercetin prevents progression of emphysema in elastase/LPS-treated mice, we examined the effects of quercetin on indices of lung oxidative stress and inflammation. Elastase/LPS-exposed mice were treated with 0.2 mg of quercetin for 10 days and lung levels of TBARS, *iNOS *mRNA and *Hmox-1 *mRNA determined. Compared to unexposed mice either treated with vehicle or quercetin, elastase/LPS-exposed mice treated with vehicle showed significantly increased levels of TBARS and *iNos*, and decreased levels of *Hmox-1 *mRNA. The ratio of *iNos/Hmox-1 *was increased (Figures 2A to 2D). In contrast, elastase/LPS exposed mice treated with 0.2 mg of quercetin for 10 days showed significantly reduced TBARS, increased *Hmox-1 *mRNA and decreased *iNos/Hmox-1 *compared to vehicle treated controls. These results indicate that exposure of mice to elastase/LPS increases oxidative stress, and that treatment with quercetin reverses this effect.

### Quercetin treatment reduces lung inflammation in elastase/LPS-exposed mice

**Figure 3:**
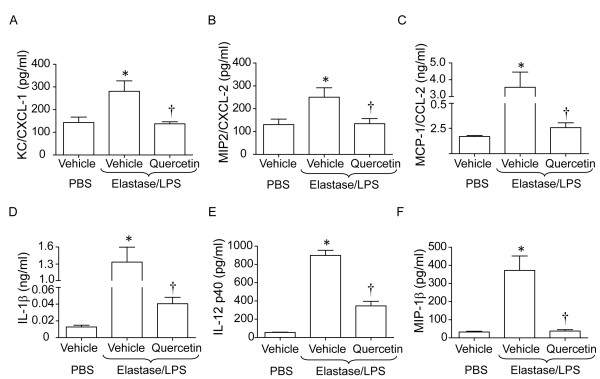
**Quercetin treatment decreases chemokine and cytokine levels in elastase/LPS exposed mice**. Mice exposed to elastase/LPS were orally gavaged with vehicle or quercetin as described. A to F. Elastase/LPS exposed mice show increased levels of KC, MIP2, MCP-1, IL-1β, IL-12p40 and MIP-1β compared to control naïve mice. Quercetin treatment reduces all the examined cytokines and chemokines. Data represent mean and SEM (n = 10, *different from PBS/vehicle group, p≤0.05; † different from vehicle-treated elastase/LPS exposed mice, p≤0.05 one-way ANOVA).

Lung cytokine levels, histology, total and differential cells counts in the BAL, and expression of the mucin gene *Muc5AC *were determined to test whether quercetin treatment reduces inflammation in elastase/LPS exposed mice. As previously noted, elastase/LPS-exposed mice showed increased protein expression of the chemokines KC/CXCL-1, MIP-2/CXCL-2 and MCP-1/CCL2 and pro-inflammatory cytokines IL-1β, IL-12p40 and MIP-1β (Figure 3). Compared to vehicle, quercetin treatment significantly decreased the levels of all chemokines and pro-inflammatory cytokines examined. PBS-exposed mice treated with quercetin showed similar levels of all cytokines measured compared to mice treated with vehicle (data not shown).

**Figure 4:**
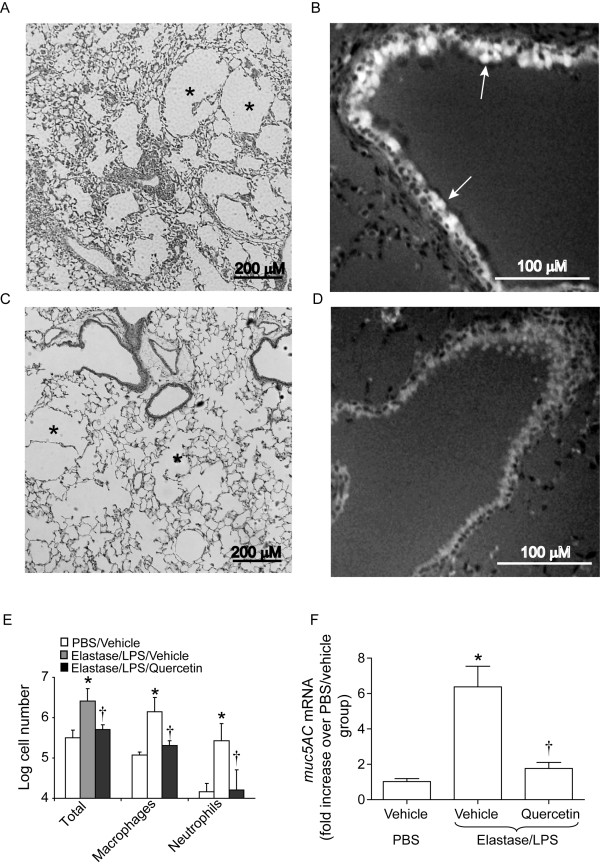
**Quercetin treatment reduces lung inflammation and reverses goblet cell metaplasia**. Lung sections from elastase/LPS exposed mice were stained with H & E or immunostained with an antibody to Muc5AC. A and B. Mice treated with vehicle show mild-to-moderate wide-spread lung inflammation, emphysema and goblet cell metaplasia. C and D. Mice treated with quercetin show less emphysema with very mild inflammation and a complete reduction in MUC5AC producing goblet cells. Asterisks in A and C represent emphysema. Arrows in B indicate MUC5AC- producing goblet cells. Images are representative of 6 mice per group. E. Examination of BAL fluid reveals increased numbers of total cells, macrophages and neutrophils in elastase/LPS treated mice, which were almost completely reversed by quercetin treatment. F. qPCR analysis of total lung RNA shows increased *Muc5AC *transcript levels in elastase/LPS-exposed mice, and this was reduced in quercetin treated mice. Data represent mean and SEM (n = 10, *different from PBS/vehicle group, p≤0.05; † different from vehicle treated elastase/LPS exposed mice, p≤0.05 one-way ANOVA).

Evaluation of H & E-stained lung sections showed wide-spread lung inflammation and emphysema in elastase/LPS exposed mice as observed previously (Figure 4A). Quercetin-treated elastase/LPS exposed mice showed an overall reduction in lung inflammation compared to vehicle treated mice (Figure 4C). Immunostaining of lung sections with anti-MUC5AC antibody showed intense signals in the airway epithelium of elastase/LPS-exposed mice treated with vehicle but not mice treated with quercetin (Figures 4B and 4D). Consistent with the histologic changes, elastase/LPS-exposed mice showed increased total cell counts, macrophages and neutrophils compared to PBS-exposed mice, and each of these variables was significantly reduced by quercetin (Figure 4E). We also observed decreased mRNA expression of Muc5AC in quercetin-treated, elastase/LPS-exposed mice compared to vehicle-treated mice (Figure 4F).

### Quercetin treatment inhibits MMP9 and MMP12 activity and increases Sirt1 expression

**Figure 5:**
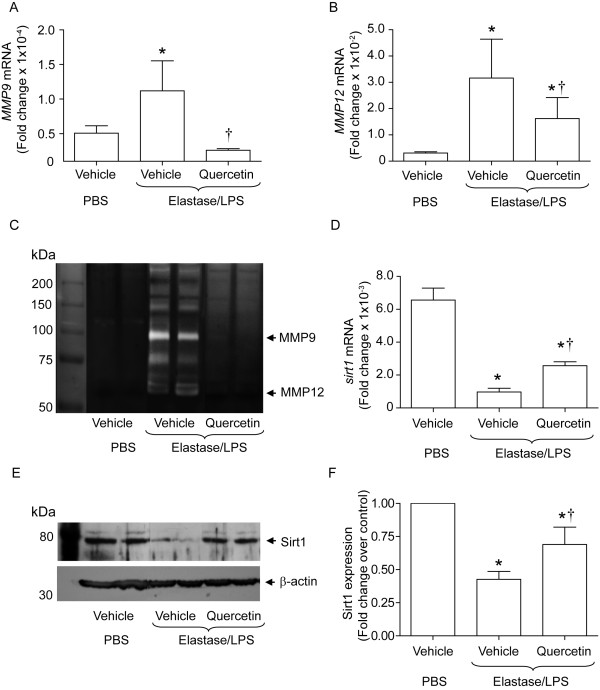
**Quercetin treatment decreases levels of MMP9 and MMP12 and increases expression of SIRT1 in elastase/LPS-exposed mice**. mRNA expression of *Mmp9*, *Mmp12*, and *Sirt1 *was measured by qPCR. MMP activity was determined by gelatin zymography. Sirt1 protein level was measured in the lung homogenates by Western blot analysis. A and B. Quercetin treated elastase/LPS exposed mice show significantly reduced *Mmp9 *and *Mmp12 *mRNA levels compared to mice treated with vehicle. C Quercetin treatment completely reduces MMP9 and MMP12 activities in elastase/LPS exposed mice. D and E. Quercetin increases Sirt1 mRNA and protein levels in elastase/LPS exposed mice. F. Ratio of Sirt1 protein/β-actin normalized to control mice calculated from 6 mice per group. Data represent mean and SEM (n = 10, *different from PBS/vehicle group, p≤0.05; † different from vehicle-treated, elastase/LPS-exposed mice, p≤0.05 one-way ANOVA). Images in C and E are representative of 4 to 6 animals per group.

Increased MMP levels are thought to play a role in the development and/or progression of emphysema in COPD patients [25-27]. Consistant with this, lungs of elastase/LPS exposed mice showed increased mRNA and activity levels of MMP9 and MMP12 compared to vehicle treated PBS-exposed mice (Figure 5A-C). Lung MMP levels did not change in quercetin treated PBS-exposed mice (data not shown). Quercetin treatment significantly decreased the mRNA and activity levels of both MMP9 and MMP12 in elastase/LPS-exposed mice.

MMP9 transcription is negatively regulated by a histone deacetylase, SIRT1 (2). We examined whether reductions in *Mmp9 *and *Mmp12 *mRNA levels were associated with increases in SIRT1 expression in quercetin-treated, elastase/LPS-exposed mice. Vehicle-treated elastase/LPS-exposed mice showed an 83.2% reduction in mRNA expression of *Sirt1 *compared to mice unexposed to elastase/LPS (Figure 5D). Similarly, we observed 53% reduction in protein levels of Sirt1 in the lungs of vehicle-treated, elastase/LPS-exposed mice (Figure 5E and 5F). Quercetin treatment of elastase/LPS exposed mice increased both Sirt1 mRNA and protein levels. These results suggest that quercetin may suppress MMP9 and MMP12 expression by increasing Sirt1 levels.

### A **Sirt1 inhibitor blocks the protective effect of quercetin**

**Figure 6:**
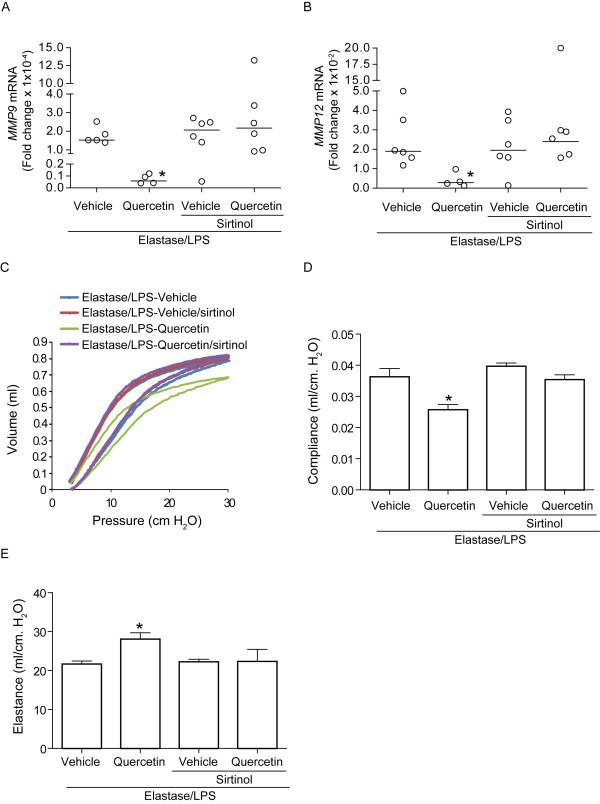
**Inhibition of Sirt1 activity in quercetin-treated mice attenuates querecetin-induced changes in MMP expression and emphysema progression**. Elastase/LPS-exposed mice were treated with vehicle or quercetin along with sirtinol or PBS. A and B. Mice treated with quercetin and sirtinol did not show reduced MMP9 or MMP12 mRNA expression. Results represent range of data with median (n = 4-6, *different from all other groups, p≤0.05; ANOVA on ranks). C-E. Sirtinol also inhibited quercetin's effects on elastic recoil (C), compliance (D) and elastance (E). Data represent mean and SEM (n = 4-6, *different from all other groups, p≤0.05; one-way ANOVA). C. Representative of 4-6 animals per group.

To determine the contribution of Sirt1 expression to the observed effects of quercetin on lung phenotype, elastase/LPS exposed mice were treated with polyethylene glycol or quercetin along with sirtinol, an inhibitor of Sirt1 activity [28]. As observed earlier, compared to vehicle-treated elastase/LPS-exposed mice, lungs of quercetin-treated mice showed reduced mRNA expression of MMP9 and MMP12 (Figure 6A and 6B) and improved elastic recoil as indicated by leftward and downward shift in pressure-volume loops, decreased static compliance and increased static elastance (Figure 6C and 6E). In contrast, mice treated with quercetin along with sirtinol showed a similar level of MMP mRNA expression as vehicle treated elastase/LPS mice. Sirtinol treatment also blocked the improvements of elastic recoil, static compliance and static elastance induced by quercetin. These results are consistent with the notion that quercetin exerts its effects by increasing Sirt1 levels, which negatively regulate MMP expression.

### Measurement of quercetin levels in plasma

Chromatographic analysis of plasma obtained from mice treated with 0.2 mg quercetin for 10 days revealed a major peak which corresponded to quercetin aglycone and other minor peaks as we observed previously [19]. Quantification of the major peak indicated a mean plasma quercetin level of 0.131 ± 0.038 μM in mice treated with quercetin. This level was significantly higher than the quercetin level observed in vehicle-treated mice (0.004 ± 0.0019 μM).

### Quercetin inhibits transcription of MMP 9 and MMP12 in alveolar macrophages *in vitro*

**Figure 7:**
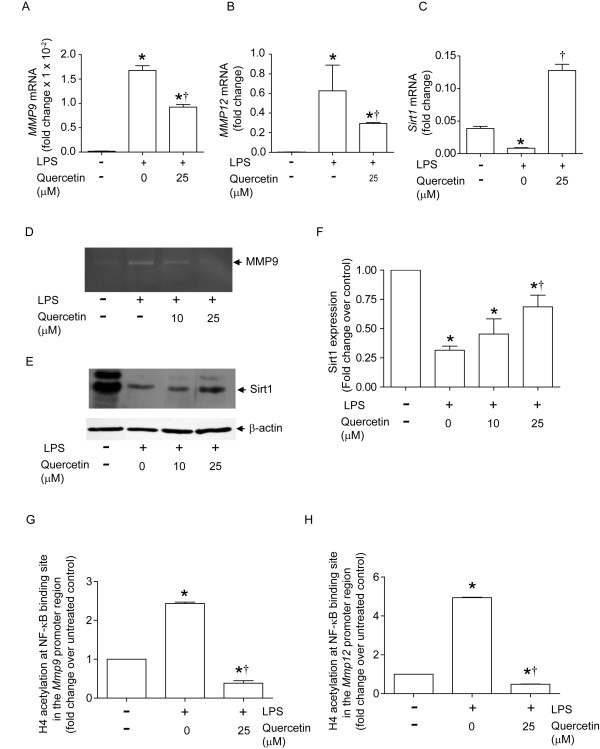
**Quercetin decreases MMP9 and MMP12 and increases Sirt1 in LPS-treated alveolar macrophages**. Mouse alveolar macrophages were treated with LPS (1 ng/ml) for 3 days and treated with DMSO (vehicle) or 25 µM quercetin for 16 h. A and B. LPS treatment induced mRNA expression of MMP9 and MMP12 which are partially reversed by quercetin. C and E. Quercetin increases Sirt1 mRNA and protein expression. D. MMP9 activity in LPS-exposed was abrogated by quercetin treatment. E. A representative Western blot showing Sirt1 and β-actin expression. F. Ratio of Sirt1 protein/β-actin normalized to untreated cells calculated from 3 independent experiments. G and H. Quercetin decreased LPS-induced histone H4 acetylation at the NF-κB binding sites in the promoter regions of both *Mmp9 *and *Mmp12*. Zymogram and immunoblot images are representative of three independent experiments. Data represent mean and SEM calculated from 3 independent experiments performed in duplicates or triplicates (*different from macrophages exposed to media in the absence of quercetin, p≤0.05; †different from LPS-exposed macrophages which are not treated with quercetin, p≤0.05 one-way ANOVA).

Sirt1, a histone deacetylase, negatively regulates MMP9 transcription by deacetylating histone H4 in the promoter region NF-κB binding site [7]. To determine whether quercetin increases H4 deacetylation at this site, we employed an *in vitro *cell culture system. Murine alveolar macrophages exposed to low levels of LPS for three days showed increased *Mmp9 *and *Mmp12 *mRNA levels, with a concomitant decrease in *Sirt1 *expression (Figure 7A-C). We also observed increased MMP9 activity (Figure 7D) and decreased SIRT1 protein expression (Figure 7E and 7F) in LPS-treated alveolar macrophages. Consistent with these *in vivo *results, *in vitro *quercetin treatment of alveolar macrophages significantly decreased LPS-induced mRNA expression of *Mmp9 *and *Mmp12 *as well as MMP9 activity, while increasing mRNA and protein expression of Sirt1. Next, we examined whether quercetin increases histone deacetylation of the MMP9 and MMP12 promoter NF-κB binding sites by ChIP assay. Histone H4 acetylation at the MMP9 and MMP12 promoter NF-κB binding sites was increased in LPS-exposed alveolar macrophages compared to media-treated controls, and this was completely inhibited by treatment with quercetin (Figures 7G and 7H). These results suggest that quercetin inhibits H4-acetylation of MMP promoters by increasing Sirt-1 expression, thereby regulating MMP expression at the transcriptional level.

## Discussion

We demonstrate that oral administration of quercetin, a major flavonoid in the human diet, significantly decreases oxidative stress and inflammation in the lungs of elastase/LPS-treated mice, which show features typical of COPD. Quercetin also decreases MMP9 and MMP12 levels by increasing expression of the type III protein deacetylase Sirt-1, a negative regulator of MMP transcription both *in vivo *and *in vitro*. Further, quercetin improves lung elasticity and compliance in elastase/LPS treated mice by preventing progression of emphysema.

In this study, mice were exposed to elastase and LPS, rather than cigarette smoke, the primary trigger of COPD in industrialized nations. This published murine model system [15] produces structural and functional features that are more pronounced and more typical of human COPD than can be achieved in wild type mice by even prolonged exposures to tobacco-smoke alone. These changes include not only pulmonary emphysema, loss of lung elastic recoil, hyperinflation, but also diffuse lung inflammation, goblet cell metaplasia, airway remodeling and markedly increased numbers of neutrophils, T and B lymphocytes, monocytes and immature macrophages in the airways and alveoli [15]. By contrast, mice exposed to cigarette smoke develop pulmonary emphysema and accumulation of alveolar macrophages, but fail to demonstrate chronic bronchitis or goblet cell metaplasia [29]. Importantly, in our model system, these morphological and inflammatory changes were accompanied by increases in lung IL-1β, IL-6, TNF-α, and MIP-2/CXCL2, markers of oxidative stress, MMP expression and reduction in Sirt1 levels, as seen in humans with COPD [30-32]. Moreover, LPS is a significant constituent of cigarette smoke [9,10]. Therefore, we believe that elastase/LPS-exposed mice are suitable for examining the therapeutic effects of quercetin or other potential drug candidates.

Epidemiologic studies of COPD patients have suggested an association of polyphenol intake with improved symptoms, as assessed by cough, sputum production, breathlessness and improved lung function, as measured by FEV_1 _[33-35]. Several *in vitro *and *in vivo *studies also have showed a direct impact of polyphenols in reducing oxidative stress and inflammation. For instance, resveratrol, a component of red wine, decreased inflammatory cytokine production from macrophages isolated from COPD patients [36] and induced synthesis of reduced glutathione by activating NF-E2-related factor (Nrf)-2, a key antioxidant transcription factor in human lung epithelial cells [37]. Curcumin, another well-studied polyphenol, has also been reported to inhibit activation of NF-κB *in vitro *and inflammation *in vivo *and restore glucocorticoid efficacy in response to oxidative stress by upregulation of HDAC2 activity in macrophages [38-40]. Curcumin also increased synthesis of Nrf2-dependent phase II antioxidant enzymes in elastase and cigarette smoke- exposed mice [41]. Quercetin, which is a potent antioxidant and possesses anti-inflammatory properties, decreased lung oxidative stress, inflammation, and prevented progression of emphysema in elastase/LPS-exposed mice.

TBARS, products of lipid peroxidation and an index of oxidative stress caused by reactive oxygen species, have been shown to be increased in COPD patients [30]. In addition, the number of HMOX-1 expressing alveolar macrophages is markedly decreased in patients with severe COPD, while iNOS expression is increased in alveolar and bronchial epithelial cells [42]. Increased staining for the nitration marker nitrotyrosine in iNOS positive cells was observed in induced sputum from patients with moderate stable COPD compared to nonsmokers [43]. Finally, overexpression of HMOX-1 in mouse lungs was shown to suppress elastase-induced emphysema by attenuating neutrophilic inflammation [44]. In the present study, quercetin treatment of elastase/LPS-exposed mice increased the expression of *Hmox-1 *and tended to decrease the expression of *iNOS*. Consistent with our observation, quercetin was noted to inhibit *iNOS *expression and NO production in LPS and interferon-γ-treated mouse BV-2 microglial cells, and this was associated with elevated expression of *Hmox-1 *[45-47]. Together, these data suggest that quercetin may inhibit chemokine and pro-inflammatory cytokine production in elastase/LPS exposed mice not only by scavenging the free radicals, but also by increasing the expression of *Hmox-1*, which suppresses *iNOS *expression [48].

In our study, quercetin treatment of elastase/LPS-exposed mice was accompanied by significant decreases in the levels of neutrophil attracting C-X-C chemokines KC and MIP-2, monocyte and macrophage chemoattractant MCP-1 and pro-inflammatory cytokines including IL-1β, IL12p40 and MIP-1β. In addition to its antioxidant effects, quercetin may attenuate lung inflammation by inhibition of protein and lipid kinases involved in inflammatory cytokine and chemokine production. Quercetin has inhibitory effects on phosphatidylinositol 3-kinase, AMP-activated kinase, casein kinase 2, p90 ribosomal protein S6 kinase, p70 ribosomal S6 kinase [49], protein kinase C [50], epidermal growth factor receptor tyrosine kinase [51] and IκB kinase [52]. Indeed, the design of the synthetic PI 3-kinase inhibitor LY294002 was based on the structure of quercetin [53].

Another important finding of this study was that quercetin inhibited MMP expression in elastase/LPS treated mice. MMP expression is increased in COPD patients and plays a critical role in development and progression of emphysema. MMP also increases mucin production, leading to airway obstruction [54-58]. Further, levels of Sirt1, a type III histone deacetylase which negatively regulates MMP9 transcription [7], were reported to be downregulated in patients with severe COPD but not in healthy smokers, suggesting a role for endogenous oxidative stress from activated neutrophils and macrophages in the reduction of Sirt1 [7]. Further, treatment of cigarette smoke-exposed mice with Sirt1 activator blocked MMP9 expression and pulmonary neutrophilic inflammation. In the present study, we found that elastase/LPS treatment increased expression and activity of MMP9 and MMP12. Interestingly, we also found decreased levels of the protein deacetylase Sirt1 in these mice. Quercetin treatment decreased MMP9 and MMP12 levels in elastase/LPS-exposed mice, while concomitantly increasing mRNA and protein levels of Sirt1, suggesting that quercetin may decrease MMP expression via deacetylation at the MMP promoter. Consistent with this, we observed that, in alveolar macrophages. quercetin decreases LPS-induced histone H4 acetylation at the MMP9 and MMP12 promoter NF-κB binding sites, thereby decreasing the transcription of MMP9 and MMP12. Furthermore, sirtinol, which inhibits Sirt1 activity, abrogated the effect of quercetin on MMP levels and lung elasticity in elastase/LPS-exposed mice. Together, these data suggest that quercetin prevents further degradation of alveolar walls by decreasing MMP expression, thereby slowing the progression of emphysema in these mice

Quercetin doses ranging between 10 to 100 mg/kg body weight have been used in previous animal studies of allergic airways disease [19,20,59]. Beneficial effects of quercetin were observed at doses as low as 10 mg/kg body weight. For example, we showed that 0.2 mg (approximately 10 mg/kg) inhibited eosinophilic inflammation and airways responsiveness in cockroach allergen-sensitized and challenged mice [19]. At this dose, quercetin levels of 0.25 μM were achieved. In the present study, plasma quercetin levels of 0.131 μM were reached. This dose was well-tolerated and was sufficient to prevent progression of emphysema. Our previous study showed that quercetin concentrations as low as 0.1 μM suppress airway epithelial cell cytokine expression *in vitro *[19]. It is also possible that enteral administration of quercetin produces sufficient gut levels to modulate lung inflammation, perhaps by altering the microbiome. Normally, human quercetin plasma concentrations are in the low nanomolar range, but upon supplementation it may increase to the high nanomolar or low micromolar range [60], suggesting that the concentration of quercetin required to prevent progression of emphysema can be achieved in humans. It is possible that absorption and availability can be further increased by using glycosylated form of quercetin [61]. These levels of quercetin were reported to be safe in humans with no adverse effects (reviewed in [62]). On the other hand, a handful of *in vitro *studies suggested that quercetin metabolites may be harmful and in fact may increase oxidative stress in lung epithelial cells [63,64]. Further studies are needed to determine the appropriate dosage and form of quercetin (glycosylated versus aglycosylated) for administration to human patients.

## Conclusions

In summary, we have demonstrated that quercetin, a plant polyphenol, reduces oxidative stress, inflammation and MMP levels in elastase/LPS treated mice which show typical features of COPD. Quercetin also prevented progression of emphysema in these mice. Even after cessation of smoking, COPD patients show progressive emphysematous changes due to persistent oxidative stress and protease burden in the airways [24]. The possibility that quercetin, which reduces inflammation and MMP levels while preventing progression of emphysema, may be beneficial in patients with COPD merits clinical testing.

## Competing interests

The authors declare that they have no competing interests.

## Authors' contributions

SG designed and performed the experiments, analyzed data and drafted the manuscript. ANF and SC and AC participated in the study design and assisted with ELISA and Western blot analysis. ATC carried out all the animal treatments, JRB determined the plasma quercetin levels. JLC, FJM, SZ and MBH participated in the review of the manuscript. US participated in study design, analysis of data and preparing the manuscript. All authors read and approved the final manuscript.
